# Description and molecular diagnosis of a new species of *Brunfelsia* (Solanaceae) from the Bolivian and Argentinean Andes

**DOI:** 10.3897/phytokeys.10.2558

**Published:** 2012-03-22

**Authors:** Natalia Filipowicz, Michael H. Nee, Susanne S. Renner

**Affiliations:** 1 Systematic Botany and Mycology, University of Munich (LMU), Menzinger-Str. 67, 80638 Munich, Germany; 2 Present address: Medical University of Gdańsk, Department of Biology and Pharmaceutical Botany, Al. Hallera 107, 80-416 Gdańsk, Poland; 3 Institute of Systematic Botany, New York Botanical Garden, Bronx, New York 10458, USA

**Keywords:** *Brunfelsia*, Argentina, Bolivia, Brazil, molecular species diagnosis, morphological description

## Abstract

*Brunfelsia plowmaniana* N.Filipowicz & M.Nee **sp. nov.**, a species from humid and cloud forests of the Bolivian and Argentinean Andes, is described and provided with a molecular diagnosis, using provisions available in the recently approved *International Code of Nomenclature for algae, fungi and plants*. Specimens belonging to the new species were previously placed in the polymorphic *Brunfelsia uniflora* (Pohl) D.Don, which a molecular phylogeny revealed as polyphyletic. Revision of numerous collections revealed clear morphological differences between the new species and *Brunfelsia uniflora*, the type locality of which is in the state of São Paulo, Brazil.

## Introduction

The genus *Brunfelsia* L. (Solanaceae) comprises ca. 50 species of shrubs and small trees in the Greater and Lesser Antilles, the eastern slopes of the Andes, and the Brazilian Shield. More than half of the species are narrowly endemic and may be adapted to special habitats. The first species of *Brunfelsia* was described in 1703 (Plumier 1703), and the first and only comprehensive treatment of the genus was carried out in the 1970s and 80s (Plowman 1978; 1979; 1998). The Brazilian species placed in *Brunfelsia* have sometimes been treated as a separate genus, *Franciscea* Pohl, but most authors chose to follow Hooker (1828), who opted for a broader concept of *Brunfelsia*. A comprehensive phylogeny of the Solanaceae firmly places *Brunfelsia* in the tribe Petunieae, together with *Bouchetia* Dunal, *Calibrachoa* La Llave & Lex., *Fabiana* Ruiz & Pav., *Hunzikeria* D’Arcy, *Leptoglossis* Benth., *Nierembergia* Ruiz & Pav. and *Plowmania* Hunz. & Subils (Olmstead et al. 2008).

One of the earliest species named is *Brunfelsia uniflora* (Pohl) D. Don, a relatively common and frequently collected species described from the vicinity of Rio de Janeiro (as *Franciscea uniflora*; Pohl 1826). When revising the abundant and heterogeneous material, Plowman (1979, 1998) chose to retain a broad concept of this species although he noted that the species had a disjunct range, occurring in rain forests of southeastern Brazil, the relatively dry coast of Venezuela, and high altitude cloud forests of the Bolivian and Argentinian Andes, and that there seemed to be morphological differences that coincided with geography (Plowman 1979, 1998). However, there was insufficient fertile material for Plowman to find a satisfactory solution before his untimely death in 1989, when material from Bolivia and northwestern Argentina was just beginning to flood into herbaria. The numbers of collections in F, MO, and NY of the Andean *Brunfelsia* species described here show this explosive increase: 1920s – 1 collection, 1970s – 4, 1980s – 9, 1990s – 15, and 2000s – 14 collections. Because of his uncertainty as to the Andean populations, (Plowman 1979, 1998) annotated *Steinbach 8345* (GH) as *Brunfelsia uniflora* in 1974, *Beck 7439* (NY) as “*Brunfelsia* sp. aff. *Brunfelsia uniflora*” in 1982, and *Vervoorst-Legname 4564* (NY) as “*Brunfelsia* sp. nov. aff *Brunfelsia uniflora*” in 1985. "All three in fact represent the new species described here.

Study of all collections from Bolivia and Argentina (plus numerous collections from Brazil) and insights from a molecular phylogeny for *Brunfelsia* (Filipowicz and Renner 2012), revealed that the Andean element is a separate species, distinct from *Brunfelsia uniflora*. We here describe the new species, named to honor Timothy Plowman, and provide information about its range, habitats, and conservation status. In addition to several morphological features that distinguish the new species from *Brunfelsia uniflora*, a molecular diagnosis based on plastid and nuclear sequences clearly differentiates the new species from all its relatives.

## Taxonomic treatment

### Brunfelsia plowmaniana

N. Filipowicz & M. Nee sp. nov.

urn:lsid:ipni.org:names:77118230-1

http://species-id.net/wiki/Brunfelsia_plowmaniana

Fig. 1

#### Molecular diagnosis.

The new species differs from all other species of *Brunfelsia* at the following nucleotide positions in the plastid *ndhF* gene, position 237: Guanine not Thymine; 270: Cytosine not Guanine; and 887: Thymine not Cytosine; and in the nuclear ITS region (ITS1 spacer, 5.8S rRNA gene, ITS2 spacer) at position 52: Guanine not Adenine; 80: Cytosine not Guanine; 215 Guanine not Cytosine; and 232: Cytosine not Guanine (Coordinates from *Nicotiana tabacum* complete chloroplast genome, GenBank accession Z00044, *ndhF* gene from 12072 to 114294, and *Nicotiana tabacum* GenBank AJ300215, ITS region) (compare Table 1).

#### Type.

Bolivia. La Paz: Prov. Inquisivi, between Yamora and Iguasani, 5 km (by air) SE of Inquisivi, 16°57'S, 67°06'W, 3100 m, 13 Jan 1989 (fl), *M. Nee 37571* (holotype: LPB; isotypes NY [01418954], AD [AD99103316], CAS [26326], CORD [00006706], F [V0093209F], G [00340084], jbsc (informal acronym for the herbarium of the Jardín Botanico de Santa Cruz, Bolivia), K [K000787830], MADw 46246, MG, MO [5752063], MY, P [00478848], SP, TEX, US [01050455], USZ [27345], WIS [v0262652WIS]).

#### Description.

Shrubs or small trees, mostly with a single stem at the base and branched only above the base, 1–4 (–10) m high, to 14 cm in diameter; bark peeling or flaking, light gray or yellow-brown. Branches with the new twigs densely pubescent with hairs to 0.3 mm long, the older branches with bark smooth, glabrous, light tan, drying in irregular longitudinal ridges or almost winged, glabrous. Internodes 4–12 mm long. Leaves scattered along the branchlets, simple, alternate, exstipulate, shiny and dark green above, chartaceous to subcoriaceous; lamina (2–) 4–9 × 1.3–3.8 cm, broadly lanceolate to obovate, narrowing abruptly to the acute to attenuate apex, the base somewhat asymmetrically cuneate to attenuate, the margin entire, slightly revolute, appearing glabrous at maturity, but often pubescent when young and then glabrescent, usually remaining sparsely pubescent on the midrib below with weak hairs to 0.3 (–0.4) mm long on the midrib and with shorter and fewer hairs on the midrib above, more rarely sparsely pubescent on the surface below and with fewer and shorter hairs above; lateral nerves 5–8 pairs, spreading, arching, adaxially often impressed, the tertiary veins raised and forming a fine reticulum when dry to nearly invisible, abaxially the lateral veins slightly raised and forming a looping interconnecting vein 2/3–3/4 of the way to the margin, the tertiary veins often not apparent; petiole 0.4–0.6 cm, pubescent with hairs to 0.3 mm long. Stomata paracytic. Flower solitary, terminal, often nodding, fragrant during the day. Pedicel (4–) 5–10 (–15) mm long in anthesis, not lengthening in fruit, articulate at the base, very sparsely glandular-pubescent, less so than the subtending stem and usually only with the shortest hairs. Calyx gamosepalous, tubular to narrowly campanulate, the tube 6–10.5 mm long, gradually narrowing to the pedicel and not sharply delimited from it, 0.9 cm diameter at summit, the lobes 5, more or less equal, (2–) 2.5–3 mm long, triangular or triangular-ovate, the margin cartilaginous, especially in fruit, striately veined, with very sparse glandular hairs on the outer surface, fewer than on the pedicel, the tube accrescent, not lengthening in fruit, but broadened and closely investing the proximal ½ of the fruit, the lobes stretched and broadened, and sometimes lengthened to 3.5–4 mm long. Corolla gamopetalous, with five subequal, broadly ovate lobes; tube 1.5–2.5 cm long, twice as long to a little more than twice as long as the calyx, more rarely only slightly longer than the calyx, erect, the limb spreading and 2–2.5 cm wide in anthesis, violet (lilac) with a pale yellowish-green throat, this raised and forming a ring, abruptly changing to violet on the lobes, with glandular hairs present on the mouth of the corolla tube, the lobes overlapping at the margins in bud, ca. 9 × 11 mm in anthesis; flower color fading to white with age. Stamens 4, didynamous, epipetalous; free part of filaments of the upper pair 2.5–4.5 mm long, those of the lower pair 0–2 mm long; anthers 4, dorsifixed, semicircular, 1 mm long, the dehiscence around the perimeter; stigma and upper two anthers visible at the mouth of the tube. Ovary bicarpellate, syncarpous, superior, ovoid, 2 mm long, glabrous, with oblique septa, ovules several per locule; style 1.5 cm long, slender, promptly deciduous; stigma clavate, bifid, 1.5 mm long. Fruit obovoid, coriaceous, capsular, 1.3–1.5 × 1.2–1.3 cm, probably green, and perhaps turning dark purple or black when ripe. Seeds ca. 9, brown, irregularly ovoid or oblong and subangular, 5.5–7 mm long, 3–3.5 mm wide and thick, the surface very minutely foveolate.

**Figure 1.**
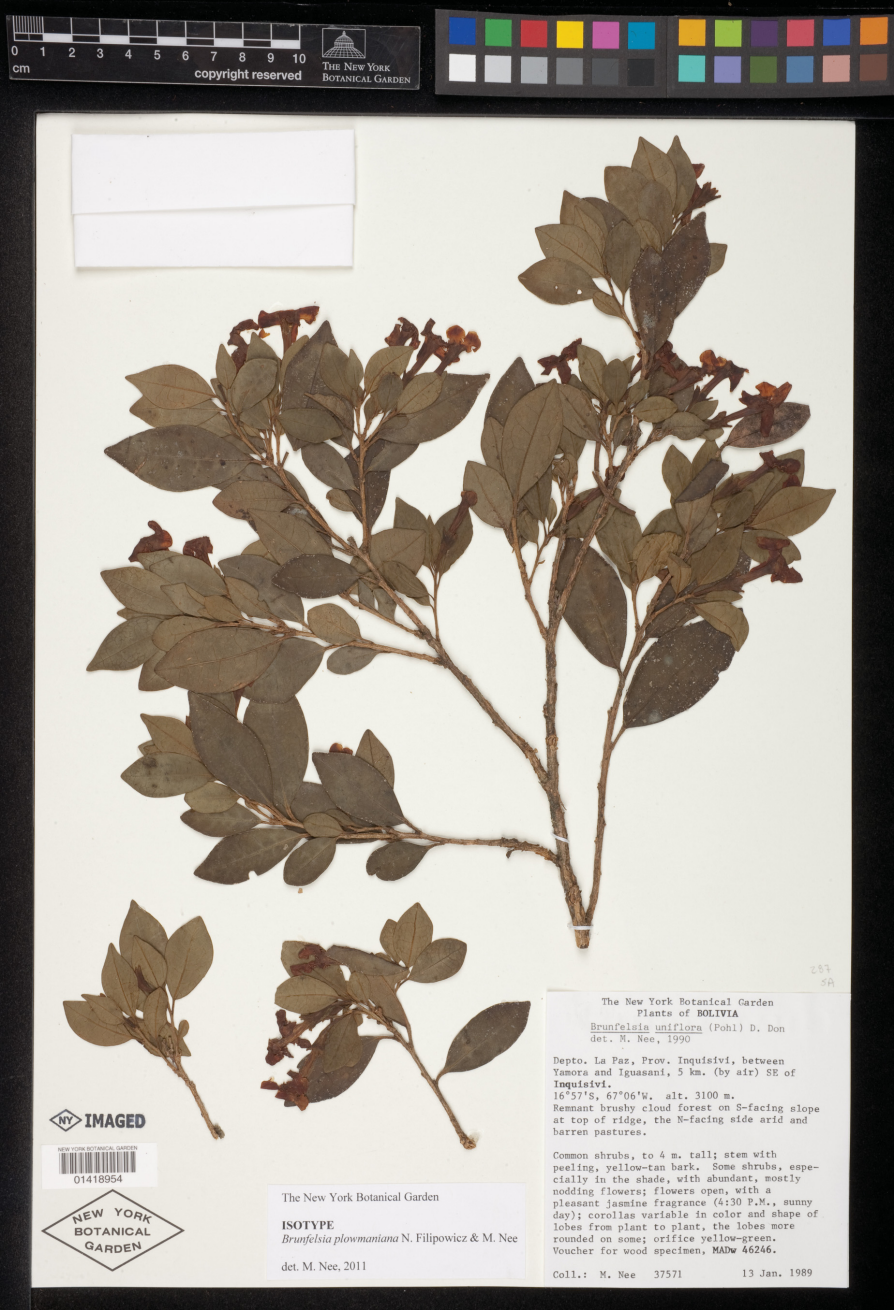
Isotype specimen of *Brunfelsia plowmaniana* N. Filipowicz & M. Nee: *M. Nee 37571* (NY).

#### Distribution

**Figure 2.**
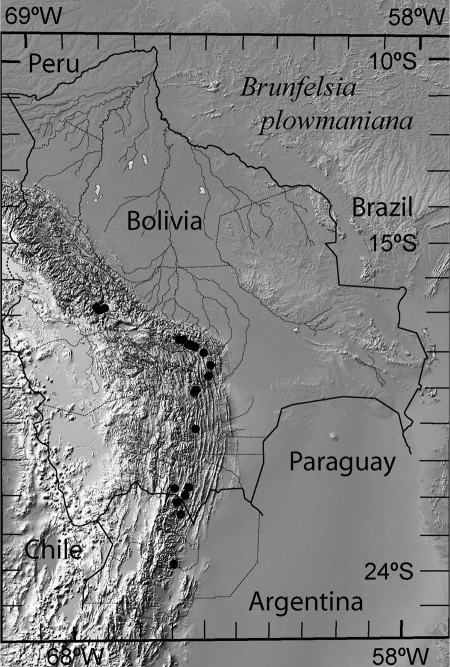
Distribution of *Brunfelsia plowmaniana* N. Filipowicz & M. Nee based on the voucher specimens listed in Taxonomic treatment and in Appendix 1.

**.** *Brunfelsia plowmaniana* is known from humid forests in the Provinces of Salta and Jujuy in northwestern Argentina, and the Departments of Santa Cruz, Cochabamba and La Paz (Prov. Inquisivi) in Bolivia. It grows at altitudes of 1500–3200 m on the predominantly N–S ridges separated by dry to arid scrub in the intervening valleys, perhaps mostly at the lower altitudinal range in the southern part of the distribution and the upper altitudinal range to the north. It has never been collected in the Provinces of Sud Yungas, Nor Yungas and Larecaja in the relatively well-explored central and northern parts of the Department of La Paz, so the northern limit of the range likely is in Prov. Inquisivi (Fig. 2).

#### Ecology.

Strictly Andean in humid or cloud forests, with *Dicksonia sellowiana* Hook. (Cyatheaceae), *Podocarpus parlatorei* Pilg., *Podocarpus rusbyi* J. Buccholz & N.E. Gray, *Prumnopitys exigua* Silba (Podocarpaceae), *Alnus acuminata* HBK. (Betulaceae), *Weinmannia* spp. (Cunoniaceae), *Blepharocalyx salicifolius* (HBK.) O. Berg, *Myrcianthes callicoma* McVaugh, *Myrcianthes pseudomato* (D. Legrand) McVaugh, *Siphoneugena occidentalis* D. Legrand (Myrtaceae), *Clethra* sp. (Clethraceae), *Viburnum* sp. (Caprifoliaceae), and numerous other Solanaceae such as *Cestrum strigilatum* Ruiz & Pav., *Lycianthes radiata* (Sendtn.) Bitter, *Saracha punctata* Ruiz & Pav., *Sessea hypotephrodes* Bitter, *Solanum aligerum* Schltdl., *Solanum aphyodendron* S. Knapp, *Solanum asperolanatum* Ruiz & Pav., *Solanum confusum* C.V.Morton, *Solanum fiebrigii* Bitter, *Solanum maturecalvans* Bitter, *Solanum saturatum* M. Nee and *Solanum stellatiglandulosum* Bitter. About one in three specimens of *Brunfelsia plowmaniana* is draped with epiphytic lichens, mosses and liverworts (including *Plagiochila* sp. and *Frullania atrata* (Sw.) Nees), reflecting the cloud forest habitat.

#### Etymology.

Named to honor the late Timothy Plowman (1944-1989), an American botanist fascinated with Neotropical plants of ethnobotanic importance, among them *Brunfelsia*.

#### Common names.

Few common names have been recorded: “bella unión” (*Nee & Vargas 38253*); “hierba mala”, “mata burro” (*Arroyo et al. 4043*). “Bella unión” is commonly used for other species of *Brunfelsia* in Bolivia (*Brunfelsia boliviana* and *Brunfelsia grandiflora*), the “beautiful union” referring to the inflorescences with both violet and white flowers together. The terms “bad herb” and “burro killer” indicates that the foliage is poisonous to grazing animals.

**Conservation status.**The species is often found in heavily grazed lower edges of the cloud forest where grazing, deforestation and fires are a threat to local populations. Even though the range is narrow, it is about 800 km long; and many populations are in undisturbed or protected areas.

#### Specimens examined

(The list of the vouchers examined with detailed locality, GPS coordinates where applicable and herbaria barcodes is presented in Appendix 1)**.** ARGENTINA. **Jujuy**: Dpto. Ledesma, 21 Oct 1979 (fl), *A.L. Cabrera et al. 30918* (F); Parque Nacional Calilegua, 19 Nov 1980 (fl), *A.L. Cabrera et al. 32115* (MO); Serranía de Calilegua, 18 Oct 1963 (fl), *H. A. Fabris 4502* (M); Parque Nacional Calilegua, 17 Dec 1998 (fr), *O. Morrone et al. 3485* (MO); Dpto. Vallegrande, 10 Oct 1969 (fl), *P.R. Legname & A.R. Cuezzo 7168* (GH). **Salta**: Dpto. Santa Victoria, Los Toldos, 2 Oct 2001 (fl), *G.E. Barboza et al. 282* (CORD); Dpto. Santa Victoria, 18 Aug 1971 (fl), F. *Vervoorst & P. R. Legname 4564* (NY); Los Toldos, 5 Sep 1979 (fr), *P.R. Legname & A. R. Cuezzo 8615* (GH); Bosque Grande, 18 Sep 1972 (fl), *L.A. Marmol et al. 9228* (GH); Los Toldos, Río Toldos, 30 Sep 1987 (fl), *L J. Novara et al. 7124* (B); Parque Nacional Baritu, 21–22 Sep 1990 (fl), *L.J. Novara 10023* (M). BOLIVIA. **Chuquisaca**: Prov. Boeto, 17 Nov 1994 (fl), *M. Serrano 1099* (NY); Prov. Siles, 11 Jan 2007 (fl), *M. Jiménez et al. 566* (NY); road to Villa Serrano, 3 Jan 1996 (fl), *J R.I. Wood 10372* (NY); Nuevo Mundo, 19 Oct 1997 (fl), *J R.I. Wood 12722* (NY); **Cochabamba**: Prov. Ayopaya, 29 Nov 1981 (fl), *S. G. Beck 7439* (F, M, MO, NY); Independencia, 7 May 1988 (fr), *S. G. Beck & R. Seidel 14442* (NY); Prov. Carrasco, 10 Feb 1987 (fr); *M. Nee & J. Solomon 34040* (F, LPB, MO, NY, TEX); “Churro”, 5 Mar 1988 (fr), *M. Nee et al. 36492* (CAS, JBSC, K, LPB, MO, NY, P, TEX); Siberia, 25 Sep 2007 (fl), *J. Terán et al. 1233* (MO). **La Paz**: Prov. Inquisivi, 13 Jan 1989 (fl), *M. Lewis 35070* (LBP, MO); Iguasani, 20 Jun 1990 (fr), *M. Lewis 37401* (LPB); Inquisivi, 30 Dec 1988 (fl), *M. Lewis 882113* (F, MO, NY); Machacamarca, 18 Mar 1988 (fl), *M. Nee 36711* (F, LPB, NY); Inquisivi, 13 Jan 1989 (fl), *M. Nee 37573* (F, LPB, MO, NY, US). **Santa Cruz**: Prov. Caballero, 17 Jun 1995 (fr); *J.R. Abbott & A. Jardim 17031* (MO, NY); Siberia, 8 Oct 2006 (fl), *L. Arroyo et al. 3569* (M, MO, NY); Enpalme, 14 Sep 2002 (fl), *A. Carrasco et al. 21* (MO, NY); Torrecillas, 17 Apr 2003 (fr), *J.A. Carrasco et al. 126* (MO); Comarapa, 26 Jun 1998 (ster), *R. Darius 28* (USZ); Carretera Fundamental 4, 9 Dec 1975 (fl), *C. Davidson 3846* (F, MO, NY); Enpalme, 7 Feb 2004 (fr), *E. Fernández et al. 2582* (MO); Parque Nacional Amboró, 17 Jun 1995 (fr); *A. Jardim et al. 1991* (MO, NY); Comarapa, 5 Nov 2003 (fl), *C G. Jordán et al. 505* (NY); 17°50'S, 64°41'W, 8 Sep 2002 (bud), *N. Ledezma et al. 62* (MO); 11 km NW Torrecillas, 15 Oct 1997 (fr), *J. Müller & J. Heinrichs 6590* (MO); vic. Tinque Laguna, 17°51'S, 64°32'W, 25 Nov 1999 (fl), *M. Nee 50606* (F, LPB, MO, NY, USZ, WIS); highway from Comarapa to Cochabamba, 5 May 2001 (fr), *M. Nee et al. 51741* (BM, LPB, NY, USZ, WIS); highway from Epizana to Comarapa, 24 May 2001 (fr), *M. Nee et al. 51857* (NY, USZ, UT); 50 km al N de Mataral, 25–26 May 1989 (fr), *D. N. Smith et al. 13343* (LPB, MO); Comarapa, 20 Oct 1928 (fl), *J. Steinbach 8345* (A, F, MO, NY); P. N. Amboró, 12–13 May 1992 (fr), *I. Vargas et al. 1341* (NY, USZ); La Siberia, 9 May 1993 (fr), *I. Vargas et al. 2379* (MO, NY, USZ); P. N. Amboró, Comarapa, 10 May 1993 (fr), *I. Vargas et al. 2399* (MO, NY, USZ); P. N. Amboró, 2400–2600 m, 18–25 Oct 1993 (fl), *I. Vargas & A. Jardim 2978* (NY), *2998* (MO, NY, USZ); Siberia, 4–6 Nov 2003 (fl), *I. G. Vargas & C. G. Jordán 7008* (MO, NY). Prov. Florida, 23 Dec 1989 (fr), *M. Nee & I. Vargas 38253* (AD, JBSC, MO, NY, US, USZ). Prov. Vallegrande, 24 Aug 2008 (fl), *L. Arroyo et al. 3963* (NY); Postrervalle, 26 Jun 1999, *B. Mostacedo & Y. Uslar 4162* (USZ). Tarija: Prov. Arce, 3 May 2005 (fl), *M. Serrano et al. 6018* (NY); Mun. Padcaya, 26 Apr 2005 (fr), *M. Serrano et al. 6288* (MO, NY); Padcaya–Motovi, 24 Sep 1927 (fl), *C. Troll 240* (B, M).

#### Discussion.

Allof the specimens from Bolivia and Argentina cited above belong to a single, morphologically uniform species that differs from *Brunfelsia uniflora*, the type of which is from eastern Brazil, in morphology as well as nuclear and plastid substitutions as specified in the molecular diagnosis and Table 1. The leaves of the Andean material are uniformly of a thicker texture, with distinct reticulate venation above; they look more like those of *Brunfelsia latifolia* (Pohl) Benth. of eastern Brazil, rather than those of Brazilian material determined as *Brunfelsia uniflora* by T. Plowman (mostly housed at F). The Brazilian material determined by Plowman is heterogeneous in indumentum; the petioles and pedicels (more particularly in the young stages) may be glabrous, very finely puberulent with tiny hairs, pubescent with weak hairs (the most like the Andean material), or pilosulose with straight hairs. The description given by Plowman (1998) has the inflorescence as “1-flowered, terminal, produced at tips of new shoots, sessile, rarely short pedunculate.” However, the protologues of *Franciscea uniflora* Pohl. and *Franciscea hopeana* Hook. both mention solitary flowers. Based on the vouchers from near the type locality, identified as *Brunfelsia uniflora*, it appears that Plowman’s description is correct, however, and this species has highly reduced cymose inflorescences with (usually) single flowers and scars visible on it. The solitary flower thus seems to be a morphological feature restricted to the Bolivian/Argentinean entity that we describe here.

**Table 1.** *Brunfelsia plowmaniana*-specific substitutions in chloroplast and nuclear DNA regions as compared to 59 other accessions representing 39 species of *Brunfelsia*.

**Position**	**Specific substitution**	**Notes**
***Plastid ndhF* gene**^1^		
237	T → G	*Brunfelsia plowmaniana*-specific
270	G → C	*Brunfelsia plowmaniana*-specific
887	C →T	*Brunfelsia plowmaniana*-specific
**Nuclear ITS1 spacer, *5.8S* rRNA gene, ITS2 spacer**^2^		
52	A → G	*Brunfelsia plowmaniana*-specific
215	C → G	*Brunfelsia plowmaniana*-specific

^1^ coordinates from *Nicotiana tabacum* complete chloroplast genome (GenBank accession no. Z00044), *ndhF* gene from 12072 to 114294 ^2 ^coordinates from *Nicotiana tabacum* AJ300215

The leaves of *Brunfelsia plowmaniana* are variable in size and shape, but usually are widest above the middle (obovate) with a rather abruptly narrowed apex (cuspidate) in the manner of many Myrtaceae. The raised and somewhat cartilaginous ring of the corolla throat is reminiscent of that of species of *Prestonia* (Apocynaceae) from the same geographic region; it probably reflects adaptation to pollinator foraging behaviour. Several collection labels mention that the corolla color changes from blue/violet to whitish while aging, which is common in the South American species of *Brunfelsia*. The notes from two vouchers (*M. Nee 37571*; *M. Nee 50606*) also mention diurnal anthesis, with a pleasant jasmine fragrance. Nothing is known directly of the pollinators of *Brunfelsia plowmaniana*, but the floral features described above are shared by other South American brunfelsias for which butterfly pollinations has been observed (Plowman, 1998).

There are no observations on fully mature fruits or dispersal of the seeds. Herbarium specimens with ripe or nearly ripe fruits always show them splitting neatly from the top about 1/3 of the way to the base into two equal valves.

**Figure 3.**
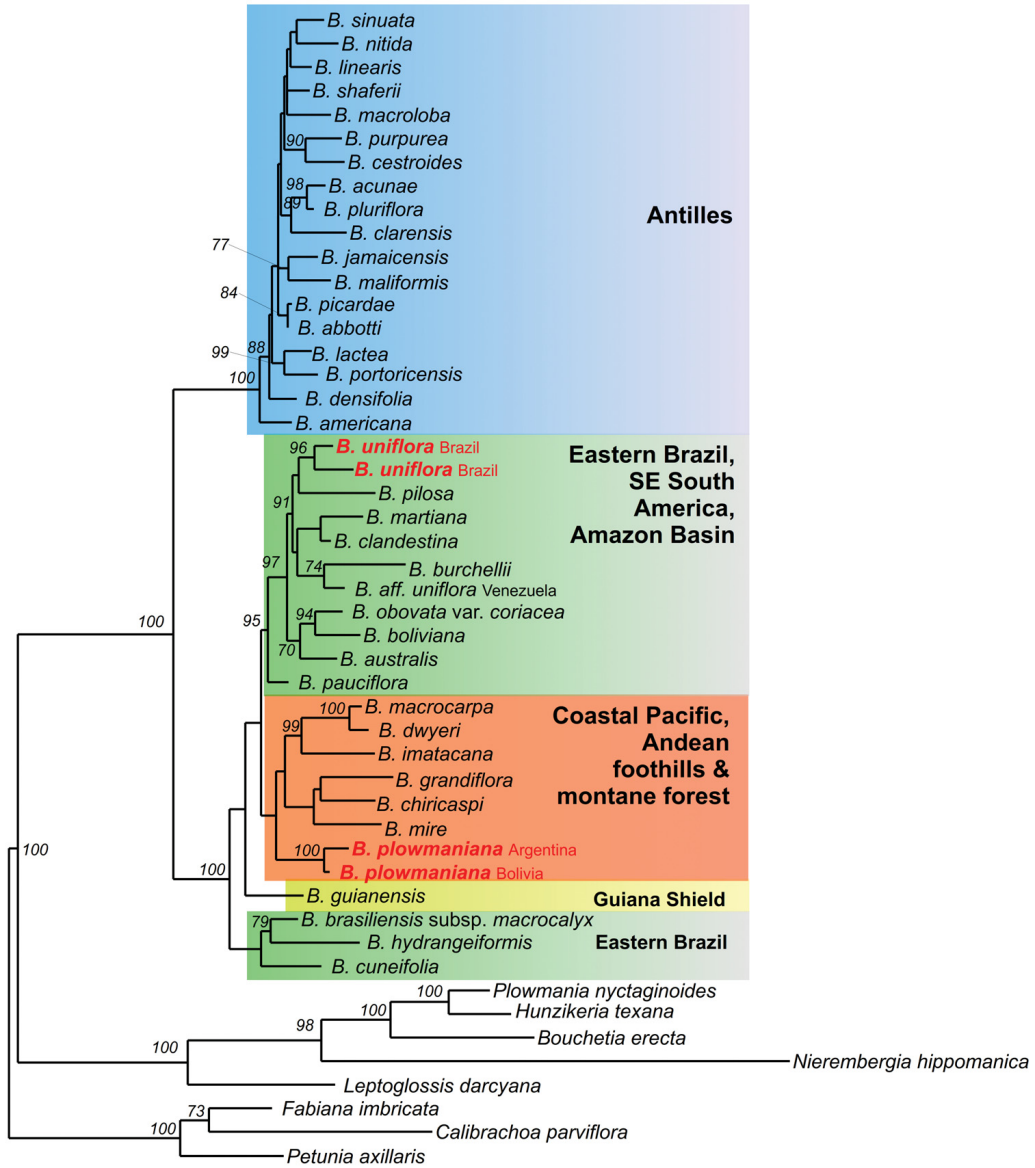
Maximum likelihood phylogram (GTR + Gamma) for a reduced dataset consisting of 41 accessions of *Brunfelsia* and 8 outgroup genera and based on the analysis of combined plastid and nuclear sequences (3784 aligned nucleotides). Numbers above branches refer to ML bootstrap support ≥70%. Placements of *Brunfelsia plowmaniana* N.Filipowicz & M.Nee and *Brunfelsia uniflora* (Pohl) D.Don (both represented by two accessions) in the tree are marked in red. Main clades are marked in different colors. For the full tree and alignment see TreeBase under acc. no. 12245.

The species that is geographically closest to *Brunfelsia plowmaniana* is *Brunfelsia boliviana* Plowman from Depto. Santa Cruz, Prov. Vallegrande south to Depto. Chuquisaca. This species has broader, thinner leaves, a corymbiform inflorescence with up to 15 flowers, a broader corolla limb, and is found in drier forest or Cháco vegetation, from a relatively narrow area in the foothills of Andes in southeastern Bolivia (up to 1200 m) (Plowman, 1981). The molecular phylogeny of *Brunfelsia* shows that *Brunfelsia boliviana* and several Amazonian species are part of a larger eastern Brazilian and Amazonian clade (see Fig. 3; Filipowicz and Renner 2012).

We initially became aware of *Brunfelsia plowmaniana* during molecular-phylogenetic work. Sequencing of 59 accessions of *Brunfelsia* representing 39 species (plus relevant outgroups) for the nuclear *ITS1*-*5.8S rRNA*-*ITS2* region, and the plastid *ndhF* gene and *trnL* intronand *trnL-F* spacer (together 3784 aligned nucleotides) revealed *Brunfelsia uniflora sensu* Plowman to be a polyphyletic entity (Fig. 3). Both *Brunfelsia uniflora* and *Brunfelsia plowmaniana* are placed in a South American group, however in distinct clades. *Brunfelsia uniflora*, represented by two accessions originating from Minas Gerais and São Paulo, belongs to a Southeastern South America and Amazon Basin clade (shaded green in Fig. 3), while *Brunfelsia plowmaniana*, represented by Argentinean and Bolivian specimens, falls in a clade from the Pacific coast and Andean region (shaded orange in Fig. 3). Molecular dating, using indirect calibration, suggests that these clades split from each other about 10 million years ago (Filipowicz and Renner 2012).

## Supplementary Material

XML Treatment for Brunfelsia plowmaniana

## Appendix 1

List of the voucher specimens included in the study. (doi: 10.3897/phytokeys.10.2558.app1) File format: MS Excel (XLS).

**Explanation note:** List of the voucher specimens used in the study with detailed locality, GPS coordinates where available and herbaria barcodes.

**Copyright notice:** This dataset is made available under the Open Database License (http://opendatacommons.org/licenses/odbl/1.0/). The Open Database License (ODbL) is a license agreement intended to allow users to freely share, modify, and use this Dataset while maintaining this same freedom for others, provided that the original source and author(s) are credited.

## Appendix 2

List of accessions included in the study. (doi: 10.3897/phytokeys.10.2558.app2) File format: MS Word document (DOC).

**Explanation note:** List of 49 accessions included in the study and used also in Filipowicz & Renner (2012) with voucher specimens, their geographic origin, and GenBank accession numbers. All the samples in the list are represented in Fig. 2. Herbarium acronyms follow Thiers (2011).

**Copyright notice:** This dataset is made available under the Open Database License (http://opendatacommons.org/licenses/odbl/1.0/). The Open Database License (ODbL) is a license agreement intended to allow users to freely share, modify, and use this Dataset while maintaining this same freedom for others, provided that the original source and author(s) are credited.

## References

[B1] Bentham G Hooker JG (1873) Genera Plantarum. Reeve & Company, 882–913.

[B2] Filipowicz N Renner SS (2012)*Brunfelsia* (Solanaceae): A genus evenly divided between South America and radiations on Cuba and other Antillean islands. Molecular Phylogeny and Evolution (accepted). doi: 10.1016/j.ympev.2012.02.026 10.1016/j.ympev.2012.02.026 22425729

[B3] Hooker WJ (1828)*Franciscea Hopeana*. Hooker’s Botanical Magazine, 55: t. 2829.

[B4] Olmstead RG Bohs L Migid HA Santiago-Valentín E García FV Collier SM (2008) A molecular phylogeny of the Solanaceae. Taxon 57: 1159–1181. http://www.ingentaconnect.com/content/iapt/tax/2008/00000057/00000004/art00010

[B5] Plowman TC (1978) In: Hawkes JG (Ed). Systematic notes on the Solanaceae.Botanical Journal of the Linnean Society 76: 294-295

[B7] Plowman TC (1979) The genus *Brunfelsia*: a conspectus of the taxonomy and biogeography. In: Hawkes JG Lester RN Skelding AD (Eds). The Biology and Taxonomy of the Solanaceae.Academic Press, London: 475-491

[B6] Plowman TC (1981) Five new species of *Brunfelsia* from South America (Solanaceae). Fieldiana: Botany, n.s. 8: 1-16

[B8] Plowman TC (1998) A revision of the South American species of *Brunfelsia* (Solanaceae). Fieldiana Bot. n.s. 39, 1–135.

[B9] Plumier C (1703) Catalogus Plantarum Americanarum. In: Boudot J (Ed) Nova Plantarum Americanarum Genera. Parisiis : Apud Joannem Boudot, Paris, 12.

[B10] Pohl JE (1826) Plantarum Brasiliae Icones et Descriptiones. Vindobonae, 1–8.

[B11] Schmidt JA (1864) Scrophulariaceae. In: Martius CPF (Ed). Flora Brasiliensis 8.R. Oldenbourg, Munich and Leipzig: 230-330

[B12] Thiers B [continuously updated] Index Herbariorum: A global directory of public herbaria and associated staff. New York Botanical Garden’s Virtual Herbarium. http://sweetgum.nybg.org/ih/

